# bFGF and collagen matrix hydrogel attenuates burn wound inflammation through activation of ERK and TRK pathway

**DOI:** 10.1038/s41598-021-82888-9

**Published:** 2021-02-08

**Authors:** Srijita Chakrabarti, Bhaskar Mazumder, Jadab Rajkonwar, Manash Pratim Pathak, Pompy Patowary, Pronobesh Chattopadhyay

**Affiliations:** 1 Defence Research Laboratory, Tezpur, Assam 784 001 India; 2 Department of Pharmaceutical Sciences, Dibrugarh University, Dibrugarh, Assam 786004 India

**Keywords:** Drug delivery, Pharmaceutics, Pharmacology, Inflammation

## Abstract

Burn injuries are most challenging to manage since it causes loss of the integrity of large portions of the skin leading to major disability or even death. Over the years, hydrogels are considered as a significant delivery system for wound treatment because of several advantages over other conventional formulations. We hypothesized that the bFGF-collagen-AgSD incorporated hydrogel formulation can accelerate the rate of burn healing in animal model and would promote fibroblast cell proliferation. Neovascularization and re-epithelialization is a hall mark of burn wound healing. In the present study, histopathological investigation and scanning electron microscopy of skin tissue of Wistar rats showed almost complete epithelialisation after 16 days in the treatment group. The developed hydrogel showed significantly accelerated wound closure compared with a standard and control group. The faster wound closure resulted from increased re-epithelialization and granulation tissue formation because of the presence of collagen and growth factor. Expressions of proteins such as TrkA, p- TrkA, ERK1/2, p-ERK1/2, NF-kβ, and p-NF-kβ involved in nerve growth factor (NGF) signalling pathway were analysed by western blot. All the findings obtained from this study indicated that the hydrogel can be considered as a promising delivery system against second degree burn by faster healing.

## Introduction

Skin, the largest organ in the body, is the protective barrier against the environmental hazards and microbial infections. Post injury complications mainly involve the loss of skin integrity followed by significant disability, superficial and septic infection or even death^1^. Burn injuries are indeed among the most challenging ones to manage. Tremendous tissue damage and significant fluid loss, resulting from burn injury, impair multiple essential functions performed by skin^2^. The process of burn healing is a complex process involving epidermal regeneration, fibroblast proliferation, neovascularization, angiogenesis etc. Previous literatures reported that controlled delivery of growth factors has been recognized as a promising way of accelerating the process of burn wound healing and promoting cell-induced skin regeneration^3–6^. Among various growth factors effective for burn healing, basic fibroblast growth factor (bFGF) is a well-known cytokine for accelerating skin regeneration^7,8^. This is known as a potent mitogen and a chemo-attractant for a wide range of cells in vivo and as a growth factor for fibroblasts and capillary endothelial cells in vitro^9^. Additionally, bFGF accelerates skin regeneration through promotion of fibroblast migration and proliferation as well as it also functions in the process of endothelial cell migration and proliferation thus, promoting angiogenesis which has a potential role in the whole healing process and helps in granulation tissue formation, re-epithelialization and remodeling^5,10–12^. Migrated fibroblasts at the wound site generate and rearrange the extracellular matrix (ECM) fibres, including collagen which is the major protein in the ECM. Collagen is a secreted product of cells that form a tissue or organ and provides strength and integrity to the dermis and other supporting tissues^13^.

The role of nerve growth factor (NGF) in the processes of inflammation and tissue repair has been studied earlier^14^. NGF is produced by many types of cells including fibroblasts and keratinocytes; and NGF produced at the wounded site regulates the wound healing^14^. Therefore, there is a possibility that proteins such as Tropomyosin-receptor kinase A (TrkA), Mitogen-activated protein kinase (MAPK) or extracellular signal-regulated kinase (ERK) involve in NGF signaling pathways may regulate the wound healing. It is well-known that ERK pathway is activated by many different stimuli including growth factors and this is a major regulator of the cell migration and proliferation^15^ whereas, TrkA or Tyrosine kinases receptors are the high-affinity cell surface receptors for many growth factors, cytokines, and hormones. Additionally, silver sulfadiazine (AgSD) has been considered as the gold standard treatment in topical burn. Therefore in the present study, bFGF, collagen and AgSD incorporated hydrogel was fabricated and it was hypothesized that this hydrogel could help in epidermal regeneration, fibroblast proliferation, and neovascularization as well as it could prevent the wound from getting infected by pathogenic microorganisms. We investigated the ability of the hydrogel to promote wound healing in vivo using a partial thickness burn wound model in Wistar rats. Further, the fibroblast cell proliferation was investigated in vitro L929 mouse fibroblast cell line.

## Results

### bFGF-collagen-AgSD hydrogel promotes faster wound healing

**Figure 1:**
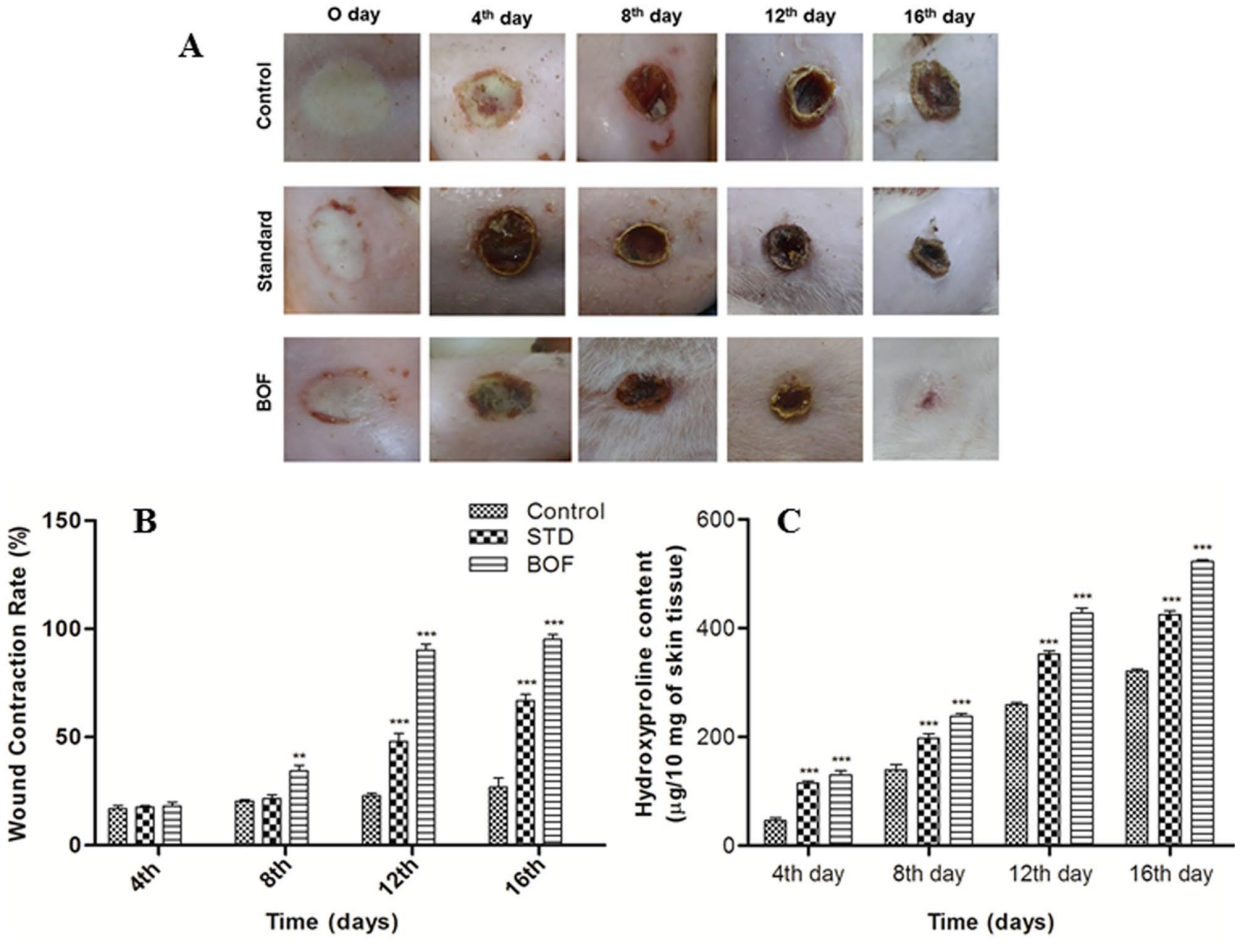
(**A**) Pictorial representation of rat skin after burn injury. (**B**) Comparative efficacy of different formulations on partial thickness burn wound healing in Wistar rats. (**C**) Hydroxyproline content of different treated groups.

To evaluate faster wound healing, wound contraction and hydroxyproline assay were carried out. Representative photographs of partial thickness burn wounds in Wistar rats have been presented in Fig. 1A. The wound contraction rates (WCR) of different groups were compared with control group on different days of treatment and were shown in Fig. 1B. No significant (p > 0.05) change was found on 4th day in each groups which means no burn wound healing was visible externally after 4 days. However, on 8th day, only BOF treated group showed significant % WCR (p˂0.001) and the % WCR of BOF treated and standard groups changed significantly (p˂0.001) on 12th and 16th day of treatment. Results were subjected to two way ANOVA followed by Bonferroni post-tests (p˂0.001) was considered significant.

It is well known that collagen helps in providing integrity to the skin tissues and promotes cellular proliferation and differentiation. Estimation of hydroxyproline, which is a major component of all types of collagen, is routinely performed to understand the progress of wound healing rate. The rate of wound healing increases when the amount of hydroxyproline increases in the connective tissue. Hydroxyproline content of the different treated groups was given in the Fig. 1C. After 16 days, the highest concentration of hydroxyproline content (524.03 ± 15.86 μg/10 mg of tissue) was found in hydrogel treated groups which also confirms the highest wound contraction rate, whereas, the untreated group has the lowest hydroxyproline content (102.30 ± 27.58 μg/10 mg of tissue) thus, the lowest wound contraction rate. Hydroxyproline content of standard group was found to be 440.04 ± 18.90 μg/10 mg of tissue. The BOF treated group has the greater hydroxyproline content than the standard group because BOF contains both collagen and bFGF which help in faster wound healing. Collagen encourages wound repopulation in cells with its regenerative potential and bFGF regulates the tissue repair process.

### bFGF-collagen-AgSD hydrogel promotes re-epithelialization, fibroblast proliferation, angiogenesis

**Figure 2:**
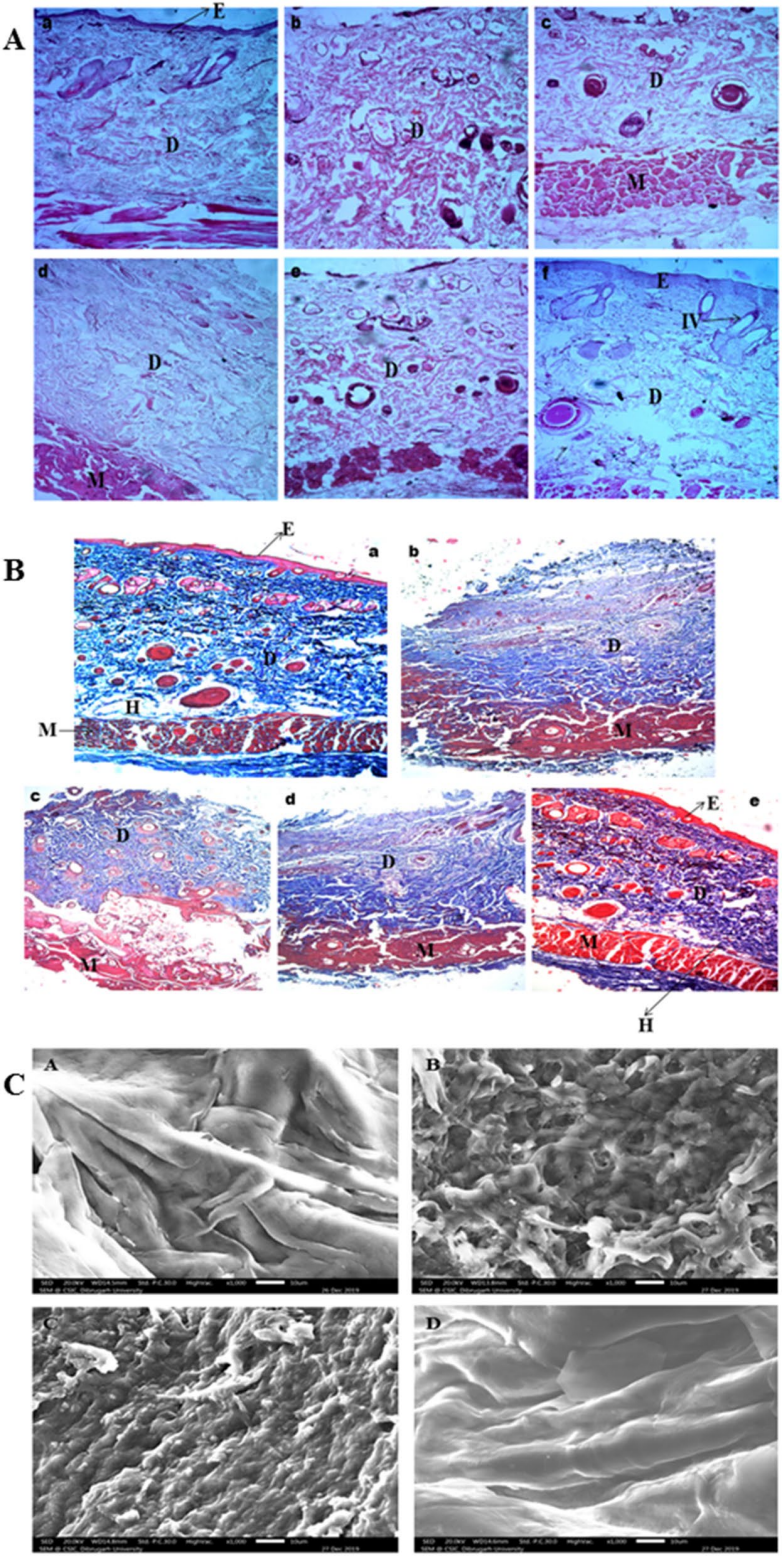
(**A**) Histopathology of different treated groups; (a) normal skin of rat, (b) untreated burned skin, (c) Rat skin of standard group on 8th day and (d) on 16th day, (e) BOF treated skin on 8th day and (f) on 16th day by using HE stain. (**B**) Histopathology of different treated groups; a) normal skin of rat, (b) untreated burned skin, (c) Rat skin of standard group on 16th day, (d) BOF treated skin on 8th day and (e) on 16th day by using MT stain. (**C**) Scanning electron microscopy of skin tissue of Wistar rats; (A) normal skin of rat, (B) untreated burned skin, (C) standard group, (D) BOF treated.

To evaluate the morphological changes of regenerating epidermis during burn healing, histopathology of skin tissues of different groups using hematoxylin–eosin (HE) and Masson-Trichome (MT) stains has been carried out (Fig. 2A,B). Further, scanning electron microscopy (SEM) of skin tissue of Wistar rats were investigated. In histopathology of skin tissues of untreated group, the epidermis was necrotic and the epithelial cells in the dermal or follicular sheath were disrupted; as a result, the surroundings of the hair follicle which contain progenitor cells could not maintain and regenerate the dermal papilla, a key component for hair growth. Thus complete loss of hair shaft follicles was observed. Moreover, disorganized extracellular matrix was found and complete debridement of epidermal tissue was also observed in partial thickness burn wounds. Burn wounds treated with hydrogel regained its normal skin structure after 16 days and animals were fully recovered with a dense and uniform neo-tissue structure, increased deposition of collagen fibres, macrophage and fibroblast proliferation, multiplication of fibrous connective tissue in the dermis, and promoted angiogenesis. In standard group, the burn wounds of animals were not fully recovered and the commercial product was unable to regain its normal skin structure. The epithelium was well-formed but with low deposition of collagen fibres. Additionally, SEM images of control rat skin section showed smooth, intact and homogeneous skin surface which is similar to previous finding^16^ whether; the morphology was changed in the untreated rat skin section as compared to the SEM image of the control rat skin (Fig. 2C). The untreated rat skin section showed irregular and discontinuous structure with loose arrangement of skin. Animals of standard group also showed irregular skin structure however, it was less discrete than the structure of untreated rat skin. Our findings concur with the observations of previous authors who investigated scanning electron microscopic characterization of wound healing of rat skin^17^ and ligament^18^. Additionally, BOF treated rat skin section showed similar surface topography as compared to the control rat skin section therefore; reformation of the skin architecture revealed that the developed hydrogel formulation helped in re-epithelialization of the burn wound rat skin which can be correlated with the histopathology findings.

### Pro and anti-inflammatory cytokines in wound repair

**Figure 3:**
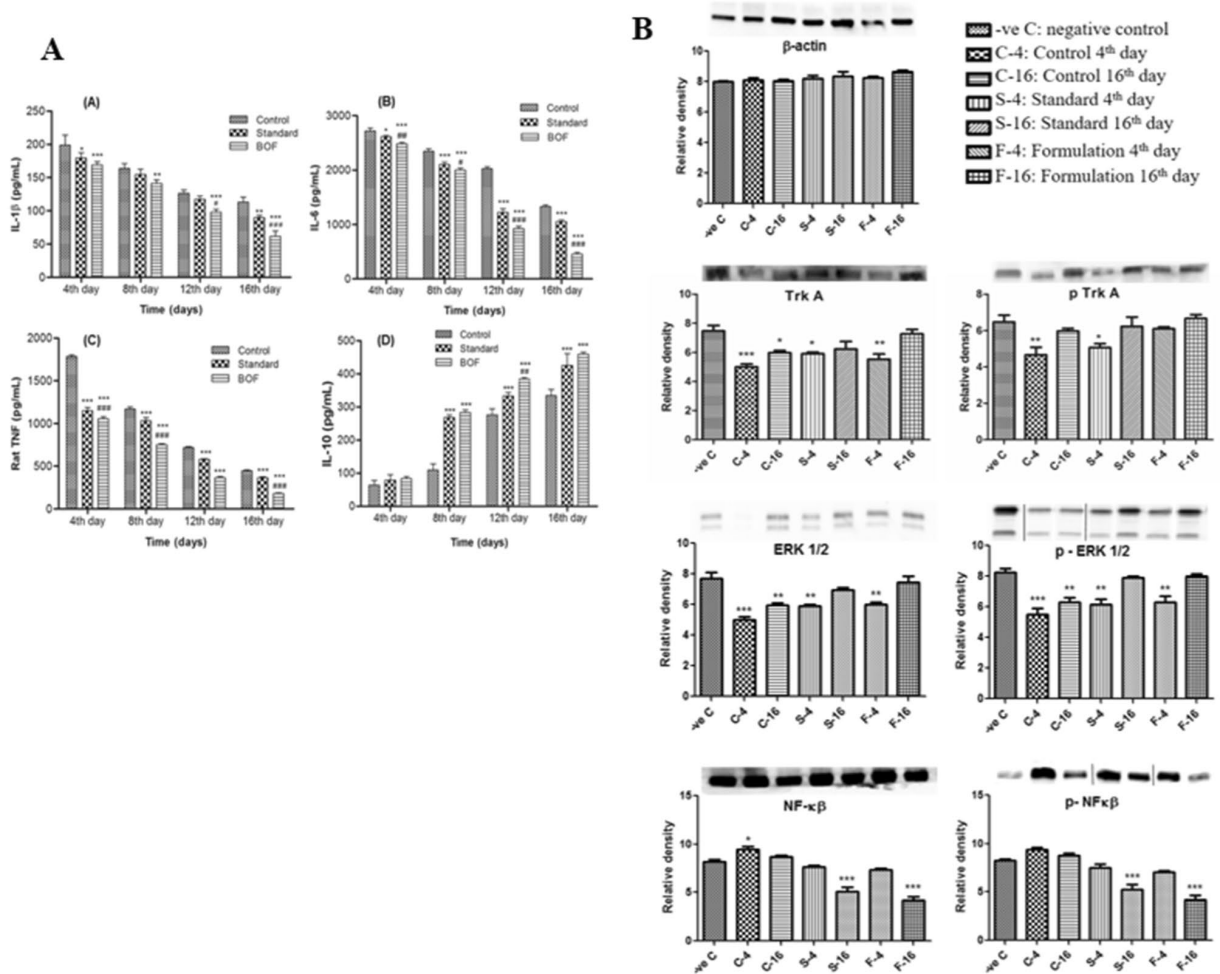
(**A**) Estimation of pro- and anti-inflammatory cytokines (A) IL-1β, (B) IL-6, (C) Rat TNF and (D) IL-10 after burn wound on 4th, 8th, 12th and 16th day by using rat serum. Data are expressed as mean ± SEM (n = 3). *** indicates p˂0.001, ** indicates p˂0.01, * indicates p˂0.05 as compared to the control group and ### indicates p˂0.001, ## indicates p˂0.01, # indicates p˂0.05 as compared to the standard group. (**B**) Western blotting analysis of β actin, Trk A, p-Trk A, ERK1/2, p-ERK1/2 NF-kβ, p-NF-kβ expressions in burn skin tissue of rats from different treatment groups. Data are expressed as mean ± SEM (n = 3). *** indicates p˂0.001, ** indicates p˂0.01, * indicates p˂0.05 as compared to the negative control group.

It has been previously reported that pro-inflammatory cytokines such as interleukins (IL)-1α, IL-1β, IL-6, and TNF-α and anti-inflammatory cytokines including IL-10 play an important role in wound repair. Pro-inflammatory cytokines influence various processes at the wound site, including stimulation of keratinocyte and fibroblast proliferation, synthesis and breakdown of extracellular matrix proteins, fibroblast chemotaxis, and regulation of the immune response^﻿4﻿^. In this present study, expressions of IL-1β, IL-6, and TNF-α were shown to be down regulated (Fig. 3A) during the inflammatory phase of healing in the BOF treated group compared to untreated group. Moreover, as reported earlier, the anti-inflammatory cytokine, IL-10, plays a major role in the limitation and termination of inflammatory responses and regulates growth and/or differentiation of various immune cells, keratinocytes and endothelial cells^﻿4﻿^. In our study, IL-10 initially increased in untreated group compared to BOF treated group and standard group. A decrease in the level of pro-inflammatory cytokines after 16 days in BOF treated group indicated that the inflammation gradually reduced; because cytokines are the primary mediators of the inflammatory reaction to burn injury^﻿19﻿^. Therefore, the use of developed hydrogel in partial thickness burn might able to reduce the inflammation.

### bFGF-collagen-AgSD hydrogel regulates burn healing via NGF signaling pathway

Previous literature suggested that topical exogenous nerve growth factor (NGF) may have roles to increase wound closure^20^. To investigate the therapeutic efficacy of the formulated hydrogel against partial thickness burn wounds, the expression levels of some proteins involve in NGF signaling pathways have been evaluated in burn granulation tissue of all treatment groups at 8th and 16th day after burn injury. We evaluated the expressions of some proteins such as Tropomyosin-receptor kinase A (TrkA), p-TrkA, Extracellular Regulated Kinase 1 and 2 (ERK1/2), p-ERK1/2, NF-kβ, and p-NF-kβ, involved in NGF signaling pathway, by western blotting. Expressions of TrkA, p-TrkA, ERK1/2, & p-ERK1/2 relative to β-actin were up-regulated and NF-kβ, p-NF-kβ were down-regulated in the treatment groups in a time dependent manner as compared to the control group (Fig. 3B). The expressions of NF-kβ and p-NF-kβ were down-regulated because it is one of the most important regulators of pro-inflammatory gene expression^21^. Further, activation of NF-kβ leads to the activation of transcription of various genes and thereby regulates inflammation^﻿22﻿^. Therefore, down-regulation of this protein indicated reduction of inflammation in burn granulation tissue. Additionally, the expressions of Trk A, p-Trk A, ERK1/2, and p-ERK1/2 were up-regulated which indicated that the developed hydrogel might activate the NGF signaling pathway and this might contribute to its ability to accelerate the rate of burn wound healing in rats. This finding is similar to a previous finding where it was stated that NGF produced in the wound may induce regeneration of fibroblasts in granulation tissue and keratinocytes at the wound edges^14^. Results were subjected to one way ANOVA followed by Dunnett’s test was considered significant. Statistical analysis was performed using GraphPad Prism statistical software version 5.01 (San Diego, California, USA).

### Bio-distribution study by in vivo imaging

**Figure 4:**
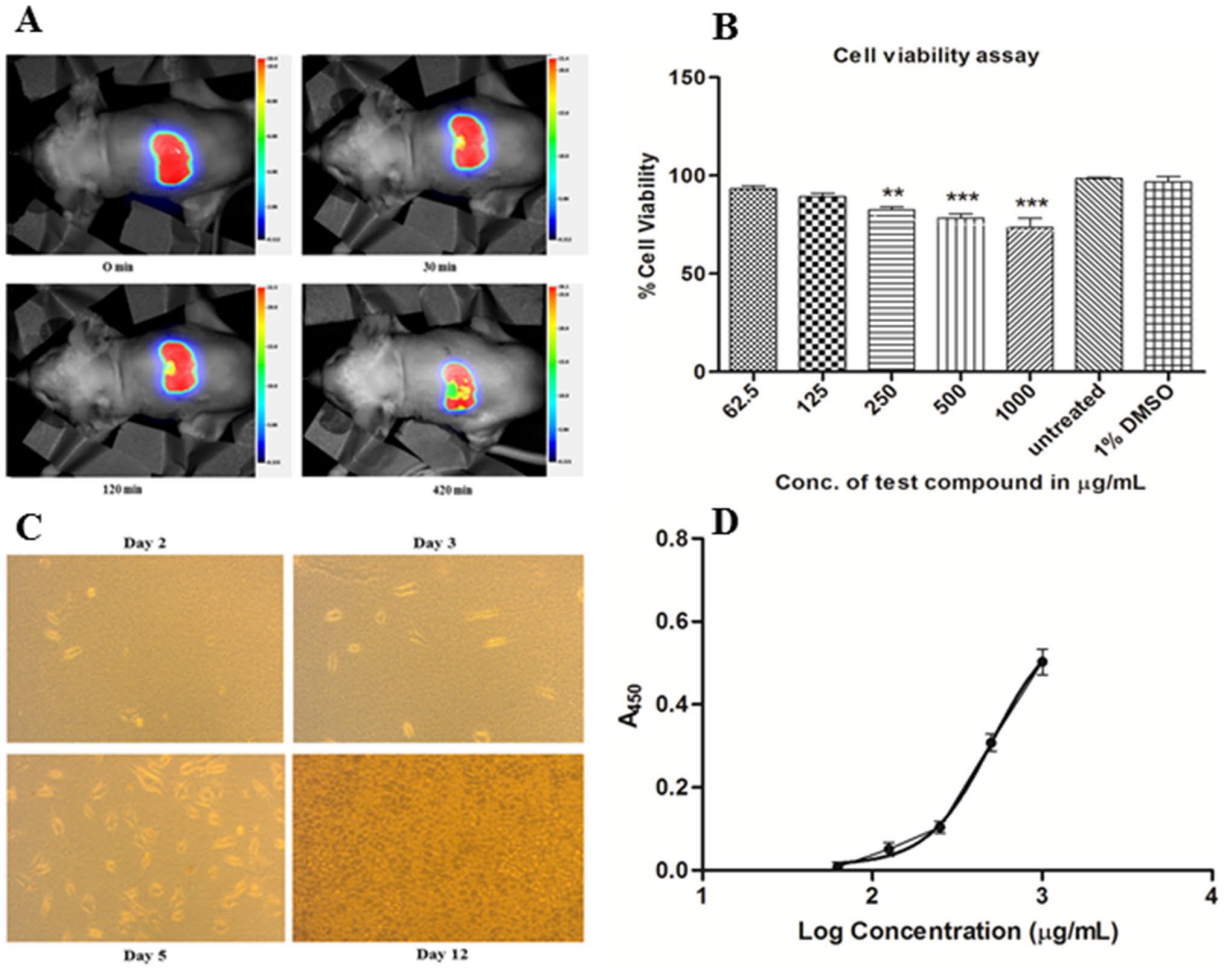
(**A**) Bio-distribution study of AgSD in partial thickness burn wounds of Wistar rats. (**B**) The cytotoxicity effect of hydrogel formulation on L929 cell line. Untreated cells were considered as negative control and 1% DMSO as vehicle control (***p˂0.0001). (**C**) Representative photographs of L929 cells maintained in culture medium. (**D**) Treatment of L929 cells with hydrogel formulation increases cell proliferation as detected by BrdU cell proliferation assay.

Representative photographs of bio-distribution of AgSD through partial thickness burn wounds of Wistar rats have been presented in Fig. 4A. After 7 hr, the intensity of the formulation was reduced but there was no distribution of AgSD into any tissues or organs which indicated that it was retained by the superficial layer of the skin. Thus the toxicity of the AgSD might be minimized and hence the adverse effects related to AgSD was reduced which is one of the important criteria of any pharmaceutical formulations. This finding is similar with the previous finding where it was stated that the absorption of AgSD was negligible through both superficial and deep dermal burn surfaces as well as through the normal skin^23^. Moreover, other previous studies about absorption of silver from partial-thickness and full-thickness burn wounds (5% body surface area) reported that most of the silver was associated with the superficial eschar and very little was absorbed into deeper layers of skin^24,25^. However, significant absorption of silver was observed from patients with extensive burns (> 40% body surface) when treated topically with AgSD, so there is the possibility of silver toxicity occurring^26–28^.

### Cytotoxicity study of hydrogel using MTT assay

Dermal toxicity of hydrogel formulation was carried out using MTT assay on mouse L929 fibroblast cell line (Fig. 4B). According to the assay, the hydrogel showed negligible cytotoxicity although cell viability reduced slightly with the increase concentration of the formulation. The cytotoxicity assay showed that % cell viability of L929 cells at the highest concentration of the formulation (1000 μg/mL) was found to be 73.42 ± 4.77. Therefore, the formulation did not inhibit cell proliferation on L929 cell line. This result indicated that the hydrogel formulation has a negligible cytotoxicity on mouse fibroblast cells and could be further assessed in burn wound. % cell viability was plotted against concentrations of test samples. Experiments were performed in triplicate and the data were presented as mean ± SD (n = 3). Further, it was evaluated by one-way analysis of variance (ANOVA) followed by Dunnett’s test. Statistical analysis was performed using GraphPad Prism statistical software version 5.01 (San Diego, California, USA).

### bFGF-collagen-AgSD hydrogel promotes fibroblast cell proliferation

Figure 4C displayed L929 cells showed active proliferation and fibroblast shaped cells after 2, 3, 5 and 12 days. BrdU cell proliferation assay was performed to investigate the cytotoxicity of AgSD or quantify the cell proliferation induced by bFGF. The result of this assay was illustrated in Fig. 4D. It showed that the absorbance at 450 nm was increased with the increasing concentration (62.5, 125, 250, 500 and 1000 μg/ml) of the hydrogel formulation which means BrdU incorporation was increased in L929 fibroblast cells treated with hydrogel formulation thus, the cell proliferation was increased. Absorbance at 450 nm (A_450_) was plotted against log concentrations of test samples. Experiments were performed in triplicate and the data were presented as mean ± SD (n = 3). Further, it was evaluated by dose response curve using GraphPad Prism statistical software version 5.01 (San Diego, California, USA).

## Discussion

The process of burn healing is a complex process involving epidermal regeneration, fibroblast proliferation, neovascularization, angiogenesis etc. Over the past few years, several investigations have been made to improve wound closure rate and healing time because there are numerous antimicrobial topical formulations which are sufficient to prevent the pathogenic infections but cannot able to achieve faster wound closure. In order to overcome these drawbacks, researchers were inspired by the concept of exogenous application of growth factors found to be promising in burn wound treatment as it may decrease the healing period^29,30^. Therefore, in the present research, AgSD and bFGF loaded collagen based hydrogel formulation has been developed in our laboratory for the first time to facilitate rapid burn wound healing simultaneously preventing pathogenic infections.

The wound contraction rate and the hydroxyproline content found maximum in the BOF treated group in comparison to untreated and standard groups. It might happen due to the presence of bFGF and collagen. bFGF promotes many cells such as dermal fibroblasts, keratinocytes, endothelial cells, melanocytes etc.; and induces tissue remodeling, wound healing and neovascularization due to its mitogenic and angiogenic characteristics^31^. Despite of several advantages including a multifunctional role in stimulation of cell growth and tissue repair; bFGF has a very short biological half-life when injected and is unstable in solution; moreover, rapid enzymatic degradation make it difficult to be applied in the free form, thus unable to achieve effective concentrations to treat wound healing^30^. Therefore, as per previous literatures, to overcome these problems, bFGF was encapsulated within collagen^32–34^. Collagen plays an important role in the formation of tissues and also can form fibers with extra strength and stability through its self-aggregation and cross-linking. Thus it can be widely used in biomedical application^35^. Additionally, histopathology and SEM images revealed that burn wounds treated with BOF regained its normal skin structure after 16 days and animals were fully recovered with re-epithelialization, fibroblast proliferation, and promoted angiogenesis. Our findings are similar to other published evidences which encourage the use of growth factors in burn healing. For example, Fu X et al., (2000) reported that the topical applications of recombinant bovine bFGF to burn wounds accelerated the rate of formation of granulation tissue as well as healing times^36^. Additionally, it was also reported that in animal systems, bFGF induces cell migration, neovascularization, and granulation tissue formation; moreover, it stimulates the healing of partial- and full thickness wounds of the skin, cornea, cartilage and brain^37,38^. Another study reported that bFGF stimulates endothelial migration, proliferation and capillary like tube formation, promoting new vessel growth in vivo and in vitro^39^. Moreover, bFGF potentiates leukocyte recruitment to inflammation sites in skin and improves dermal wound healing outcomes upon direct delivery, either through a targeted peptide delivery system, or alongside tissue engineering scaffold. It has also been reported earlier that for partial-thickness skin defect such as second-degree burn, bFGF should be started at the earliest possible after such an injury^31^.

The pro-inflammatory cytokines such as IL-1β, IL-6, and TNF-α were found down regulated during the inflammatory phase of healing in the BOF treated group compared to untreated group. A decrease in the level of pro-inflammatory cytokines after 16 days in BOF treated group indicated that the inflammation gradually reduced; because cytokines are the primary mediators of the inflammatory reaction to burn injury^19^. Therefore, the use of bFGF-collagen-AgSD hydrogel in partial thickness burn might able to reduce the inflammation.

In western blotting, the expression levels of various proteins involve in NGF signaling pathways have been assessed in burn granulation tissue. The expressions of NF-kβ and p-NF-kβ were down-regulated in the treatment groups in a time dependent manner as compared to the control group because it is one of the most important regulators of pro-inflammatory gene expression^21^. Activation of NF-kβ leads to the activation of transcription of various genes and thereby regulates inflammation^22^. Therefore, down-regulation of this protein indicated reduction of inflammation in burn granulation tissue. Additionally, the expressions of proteins such as Trk A, p-Trk A, ERK1/2, and p-ERK1/2 were up-regulated which indicated that the developed hydrogel might activate the NGF signaling pathway and this might contribute to its ability to accelerate the rate of burn wound healing in rats. As NGF produced in the wound may induce regeneration of fibroblasts in granulation tissue and keratinocytes at the wound edges^14^.

In bio-distribution study, no distribution of AgSD was found into any tissues or organs which indicated that it was retained by the superficial layer of the skin. Thus the toxicity of the AgSD might be minimized and hence the adverse effects related to AgSD was reduced which is one of the important criteria of any pharmaceutical formulations.

Further, the cytotoxic effect of the hydrogel formulation was investigated on dermal cells using MTT assay. It is well-known that MTT assay is based on the reduction of yellow tetrazolium MTT to a purple formazan dye by mitochondrial succinate dehydrogenase^40^. A typical wound healing process encompasses complex cellular changes that include inflammation, re-epithelialization, angiogenesis, granulation tissue formation, migration and proliferation of keratinocytes and fibroblasts and remodeling of ECM^41^. In the early stages of wound healing, fibroblasts play an important role by actively proliferating, migrating to wound area and transforming into myofibroblasts which facilitate wound contraction process^42^. Additionally, fibroblasts are involved in the synthesis of ECM components, immature collagen, developing mechanical forces and remodeling the scar^43,44^. The hydrogel showed minimal toxicity on L929 mouse fibroblast cell line indicating negligible cytotoxic effect of the developed formulation on dermal cells. Since the developed formulation contains bFGF along with AgSD, it may be possible that the bFGF may have negated the cytotoxic effect of AgSD. This can be supported by the findings of McCauley et al.^44^ who reported that growth factors including bFGF may have a role of cyto-protection to cells responsible for wound healing and help in initiation and modulation of the process of wound healing^45^. Further, treatment of L929 cells with hydrogel formulation increases cell proliferation as detected by BrdU cell proliferation assay and it may be possible that the bFGF may increase the proliferation because of its role in cell proliferation. As per previous literatures, bFGF is an important member of a heparin-binding protein family, which controls the proliferation, differentiation, and migration of different cells. Additionally, it is a potent regulator of cell proliferation, differentiation and function and is critically important in normal development, tissue maintenance, wound repair and angiogenesis^5,12,46^.

Finally, from the present findings it can be concluded that the developed hydrogel formulation is not cytotoxic and helps in cell proliferation however, this claim is limited to the highest concentration of the formulation and the specific cell line used in this study. Hence, the formulation needs to be studied further in partial thickness burn wound using different cell lines with higher concentrations. However, our study is one step forward to the application of this product in burn wound care.

## Materials and methods

### Study design

We hypothesized that the bFGF-collagen-AgSD incorporated hydrogel formulation would accelerate the rate of burn healing in animal model and would promote fibroblast cell proliferation as well as prevent the wound from getting infected by pathogenic microorganisms. The experimental ranges were selected on the basis of previous findings^30,47,48^.

### Animals, burn model and treatment

Healthy, adult Wistar albino rats were obtained from Central Animal facility, Defence Research Laboratory, Tezpur, Assam, India. Animals were kept in polypropylene cage providing free access of water and standard rodent chow (Pranav Agro Industries Limited, Sangli, Maharashtra, India) ad libitum. General conditions of captivity were maintained in simulated atmospheric conditions 25 (± 2) ˚C temperature; 70% RH; 12 h light/dark cycle. All experimenting protocols using animal were performed according to the “Principles of Laboratory Animal care” (NIH publication 85–23, revised 1985) and approved by the Institutional Animal Ethical Committee (IAEC) of Defence Research Laboratory, Tezpur, Assam, India (approval no. IAEC/DRL/05/2016).

The required amount of collagen, PVA, AgSD, and bFGF were added in phosphate buffer solution (PBS, pH—7.4) followed by thorough mixing with the help of magnetic stirrer^49,50^. The temperature was maintained at 4ºC during the preparation and the pH of the formulation was adjusted to 5.0.

Animals were divided into three groups i.e. control (burned but untreated), standard (burned and treated by commercial product; 1% w/w silver sulfadiazine and 0.2% w/w Chlorhexidine gluconate), and BOF (best optimized hydrogel formulation) treated group (burned and treated with bFGF-collagen-AgSD hydrogel). All rats were anaesthetized with sodium phenobarbitone. Then the trauma was performed by holding a cylindrical shaped bar with the radius of 1 cm on the shaved back skin of rats and hot water (98 ± 1ºC) was poured into this bar and held for 15 secs^51^. After the formation of burns, the rats were housed individually and treatments were given once daily until complete epithelialization. The progressive changes of burned area were photographed on day 0, 4th day, 8th day, 12th day, and 16th day. Then all images were evaluated by using size analysis software- Image J as indicated in the Results and figures.

## Supplementary Information

Supplementary Information 1.
