# Supplementary material for: bFGF and collagen matrix hydrogel attenuates burn wound inflammation through activation of ERK and TRK pathway
Source: Sci Rep. 2021 Feb 8;11:3357. doi: 10.1038/s41598-021-82888-9 (PMC7870886; doi:10.1038/s41598-021-82888-9)
Supplement: Supplementary file 1 — Supplementary Information 1. [file 41598_2021_82888_MOESM1_ESM.pdf]

<u>Supplementary file</u>

bFGF and collagen matrix hydrogel attenuates burn wound inflammation through activation of ERK and TRK pathway

Srijita Chakrabarti[a,b], Bhaskar Mazumder[b], Jadab Rajkonwar [a], Manash Pratim Pathak[a], Pompy Patowary[a], Pronobesh Chattopadhyay[a*]

[a]*Defence Research Laboratory, Tezpur, Assam- 784 001, India.*

[b]*Department of Pharmaceutical Sciences, Dibrugarh University, Dibrugarh, Assam- 786004, India.*

* Correspondence

Defence Research Laboratory, Tezpur, Assam- 784 001, India.

Tel: +91-3712258836/258508, Fax: +91-3712258534

Email: chattopadhyay.drl@gmail.com

Wound contraction rate

The progressive changes of burned area were photographed on day 0, 4th day, 8th day, 12th day, and 16th day. All burn wound images were evaluated by using size analysis software-Image J. The changes in the burn wound size were expressed as percentage contraction of the original wound size (0 day).

Percentage (%) wound contraction was calculated using following formula:

$$[(A_o - A_n)/Ao]\ X\ 100 \ldots\ldots\ldots\ldots\ldots\ldots\ldots\ldots\ldots\ (1)$$

Where, A_o wound area at 0 day and A_n is the wound area at n^{th} day.

Determination of Hydroxyproline content

As hydroxyproline is a major component of collagen, therefore, the measurement of hydroxyproline can be used as an indicator of collagen content. Hydroxyproline content was analyzed on the 4th, 8th, 12th, and 16th day as described by Neuman and Logan (54). For protein hydrolysate preparation, skin tissues were hydrolyzed in 6 N HCl for 24 h at 110 °C in tightly capped glass bottle. After neutralization of pH, the hydrolyzed samples were mixed with 1 ml of 0.01 M $CuSO_4$ followed by the addition of 1 ml of 2.5 N NaOH and 1 ml of 6% H_2O_2. The solution were then mixed properly and shaken occasionally for 5 min. All the glass bottles were incubated at 80 °C for 5 min with frequent vigorous shaking followed by cooling of the samples. Then 4 ml of 3 N H_2SO_4 was added with agitation. Finally, 2 ml of 5% p-dimethyl-aminobenzaldehyde was added to the mixture and incubated at 70 °C for 15 min and then cool the tubes by placing them in water at 20°C. The absorbance was measured at 500 nm using a UV-visible spectrophotometer. The standard calibration curve was plotted for pure hydroxyproline and used for estimation of the test samples.

Histopathological investigation

On 8th and 16th day, all rats were humanly sacrificed and burn wound granulation tissues were collected from the skin of rats to study the morphology of skin. All the tissue specimens were fixed in formalin (10%), then washed with tap water and subjected to dehydration by using different grades (70%, 80%, 90%, and 100%) of ethanol. Dehydration was followed by clearing the samples in xylene. The samples were then impregnated with molten paraffin wax, embedded, and finally blocked out. Tissue was sectioned (8 µm thick) perpendicular by using fully automated microtome-1010-SMT-118 (spencers company) and the obtained tissue sections were collected on glass slides, deparaffinised, then stained with hematoxylin-eosin (HE) and Masson-Trichome (MT) according to standard protocol. Then the stained tissue sections were placed on a glass slide mounted with a cover slip; observed and photographs of different resolution (5X and 20X) were taken using a phase contrast microscope (Axio Scope,

Carl Zeiss, Oberkochen, Germany). Finally, the microscopic images of each group were compared.

Scanning electron microscopy of skin tissue of Wistar rats

The scanning electron microscopy of skin tissue of Wistar rats provides a convenient means of examining microscopic surface topography of skin tissue (55). Skin tissues of rats were collected for scanning electron microscopy and fixed in 2.5% gluteraldehyde for 1 day (56). Then tissues were rinsed in 0.2 M cacodylate buffer, followed by fixation in 1% osmium tetroxide (OsO_4) in 0.1 M cacodylate buffer for at least 2 h at room temperature, and dehydrated through graded ethanol and dried. The dried samples were mounted on SEM mounting blocks and coated with gold using sputter coater to prevent charging. Finally, skin sections were observed under a scanning electron microscope (JSM-IT 300LV, JEOL, India Pvt. Ltd.) and images were captured at a magnification of 1000X. Scanning electron microscopic findings were correlated with histological observations.

Pro and anti- inflammatory cytokines study

In the present study serum cytokines were determined by ELISA technique. Blood was collected from all rats in serum clot activator tube (Peerless Biotech Pvt Ltd) for cytokines study and was kept at room temperature for clotting. Then, blood samples were centrifuged at 3000 rpm for 10 min within 1h after collection and then the serum was separated and stored at -80°C until assayed. The assay was performed according to the manufacturer's instructions.

Western blotting

The collected burned tissues were homogenized in RIPA buffer (Sigma-Aldrich, St. Louis, USA) and the homogenate were centrifuged at 10,000g for 10 min at 4°C. Protein concentration was estimated using the protein estimation kit (Biorad laboratories, Inc.), and quantified by measuring the absorption at 750 nm in a microplate reader (SpectraMax Plus 384, Molecular Devices, San Jose, California, USA). Equal amount of proteins were loaded into the 10% SDS-PAGE with 4% stacking gel wells and with molecular weight marker followed by electrophoresis at 110 V for 60 min; thereafter, proteins were transblotted onto the PVDF membrane. After blocking, the membranes were incubated with primary antibodies for overnight followed by 1h incubation with secondary antibodies on dancing shaker after washing with TBST. Then the membrane was incubated with ECL substrate according to the manufacturer's recommendation followed by capture the Chemiluminescent signals. Finally, the desired proteins were detected by analysing the band intensity of the target protein. During the experiment, the blots were cut into two pieces as per the molecular weight of the

primary antibodies prior to hybridization with antibodies to save the consumables. Therefore, images of all blots have been included in the supplementary file as they were, instead of images showing full length membranes with membrane edges visible.

The effect of the formulation against burn healing was studied by measuring various protein expressions such as Tropomyosin-receptor kinase A (TrkA) (1:1000, SC-80398), phosphorylated TrkA (1:1000, SC-8058), Extracellular Regulated Kinase 1 and 2 (ERK1/2) (p44/42 ERK1/2, 1:1000, SC-514302), phosphorylated ERK1/2 (pERK1/2, 1:2000, SC-136521), NF-kβ (1:1000, CST), and phosphorylated NF-kβ (1:1000, CST) involved in nerve growth factor (NGF) signalling pathway.

Bio-distribution study by in vivo imaging

Bio-distribution study was performed by *in vivo* imaging to determine the distribution of AgSD in Wistar rat. The animal was anesthetized by inhalation with 3% isoflurane (ISOTROY® 250, Troikaa Pharmaceuticals Ltd, Gujarat, India) and 1.5 L/min (45-55psi) oxygen for 5-10 min and the anaesthetized condition was maintained inside the imaging chamber throughout the period with 1.5% isoflurane. A required amount of the AgSD incorporated hydrogel was tagged with IRDye® 800CW 2-DG Optical Probe (Li-Cor, Nebraska, USA) and applied topically in the dorsal region of the anesthetized burned rat. Animal was placed in *in-vivo* imaging system and the images were acquired at different time intervals upto 420 minutes using Pearl®TRILOGY (Li-Cor, Lincoln, NE, USA) at near-infrared wavelength.. The emitted light intensity was quantified using Image Studio Software by LI-COR™.

Cell culture and maintenance

The L929 fibroblast cell line was purchased from NCCS, Pune and maintained according to the supplier's instruction. Briefly, the growth medium was removed from the flask and cells were washed twice with trypsin phosphate versene glucose (TPVG). Then TPVG was removed from the flask leaving enough so that a thin film was formed over the cell sheet. The flask was kept in horizontal position for some time followed by tapping against palm of hand and cells came off substratum. Finally, cells were aspirated with fresh DMEM supplemented with 10% FBS and transferred into a new flask. Then after 3 days cells were sub-cultured in fresh medium with 100 U/mL of penicillin, and 100 mg/mL of streptomycin at 37°C in a 5% CO_2 incubator. Cells showed specific spindle shape morphology were photographed by using inverted microscope (Axio Scope, Carl Zeiss, Oberkochen, Germany).

Evaluation of cytotoxicity by MTT assay

The cytotoxicity effect of the hydrogel formulation was determined in L929 fibroblast cell line by using MTT assay kit (EZcount^{TM} MTT Cell Assay Kit, HiMedia, Mumbai, Maharashtra, India) as per manufacturer's instructions. The L929 cell line was harvested, centrifuged and resuspended in DMEM supplemented with 10% FBS. The cell population was adjusted to 1×10^3 cells/ml, with media and then 100 µl of cell suspension were taken in each well of a 96 well plate and incubated at 37ºC in humidified 5% CO_2 incubator. After 24 h treatment, 10 µl of MTT (3-[4, 5- dimethylthiazol-2-yl]-2, 5-diphenyl tetrazolium bromide) was added to each well and the plate was wrapped with aluminium foil to avoid exposure to light. After incubation of 4 h at 37°C, when the purple precipitate was clearly visible under the microscope, 100µl of solubilization solution was added to the wells and stirred gently to enhance dissolution of the formazan crystals. Then absorbance was measured at 570 nm by using a Microplate reader (Spectramax Plus384; Molecular Devices, Sunnyvale, CA, USA). Each sample was assayed in triplicate and the percentage cell viability was calculated by the following formula as described elsewhere (57).

$$\% \text{ Cell viability} = \frac{(\text{Absorbance treated cells} - \text{absorbance blank})}{(\text{Absorbance control cells} - \text{absorbance blank}} \times 100 \ldots\ldots\ldots (2)$$

BrdU cell proliferation assay

The proliferation of L929 cells was confirmed by using 5-bromo-2-deoxyuridine (BrdU) enzyme linked immunosorbent assay kit (BioVision, CA, USA). BrdU, a pyrimidine analog, gets incorporated into the newly synthesized DNA of proliferating cells in place of thymidine. This highly sensitive, non-radioactive kit detects as less as 50-100 proliferating cells only and not the seeded cells. At first, cells were seeded in a 96-well plate and incubate for required time period then BrdU solution was added into desired wells and incubated at 37°C. Denaturing solution was then added into each well then incubated at RT for 1 hr with gentle shaking. An anti-mouse HRP-linked secondary antibody solution was added into each well followed by addition of TMB (a HRP substrate) and measured the absorbance at 650 nm for 5-30 min at RT to monitor the color development. The extent of color development is proportional to the quantity of BrdU incorporated into the cells and can be used directly as an indicator of cell proliferation. Then stop solution was added into each well to stop the color development and absorbance was measured at 450 nm by using a Microplate reader (Spectramax Plus384; Molecular Devices, Sunnyvale, CA, USA).

References (Supplementary)

1. Neuman, R.E. and Logan, M.A., 1950. The determination of hydroxyproline. J Biol Chem, 184(1), pp.299-306.

2. Brown, I. A. Scanning electron microscopy of human dermal fibrous tissue. *J Anat*. **113**, 159 (1972).
3. Provenzano, P. P., Hurschler, C. & Vanderby, R. Microstructural morphology in the transition region between scar and intact residual segments of a healing rat medial collateral ligament. *Connect. Tissue Res*. **42**, 123-133 (2001).
4. Bolla, S.R., Al-Subaie, A.M., Al-Jindan, R.Y., Balakrishna, J.P., Ravi, P.K., Veeraraghavan, V.P., Pillai, A.A., Gollapalli, S.S.R., Joseph, J.P. and Surapaneni, K.M., 2019. In vitro wound healing potency of methanolic leaf extract of Aristolochia saccata is possibly mediated by its stimulatory effect on collagen-1 expression. Heliyon, 5(5), p.e01648.

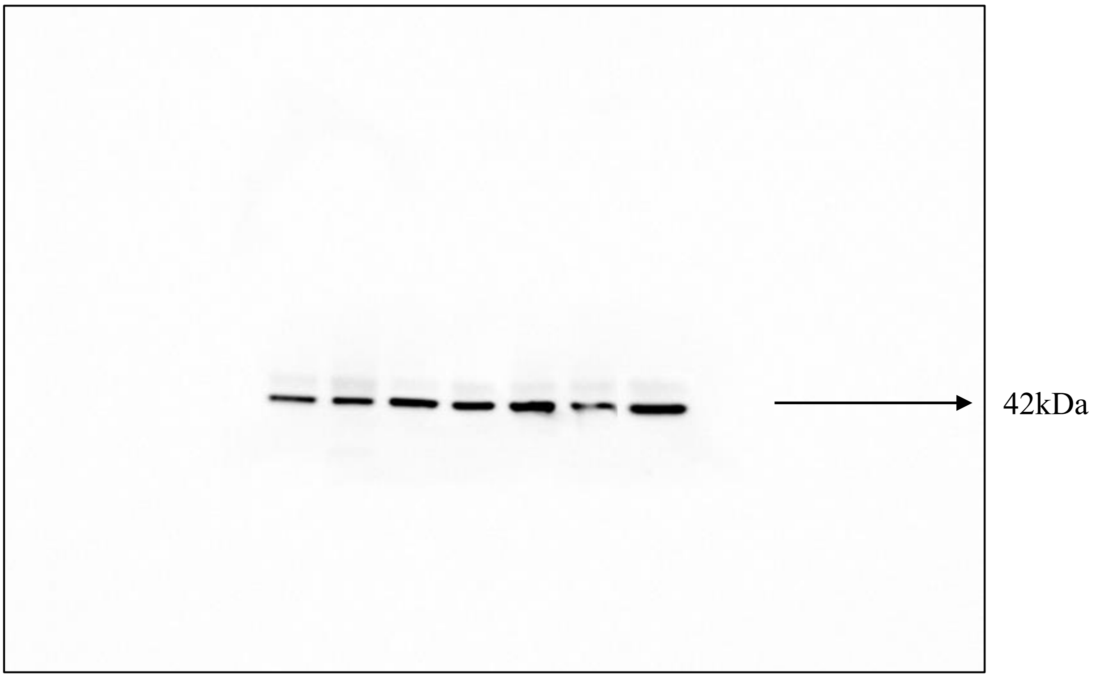

Figure 1: Full-length blots/gels of β actin

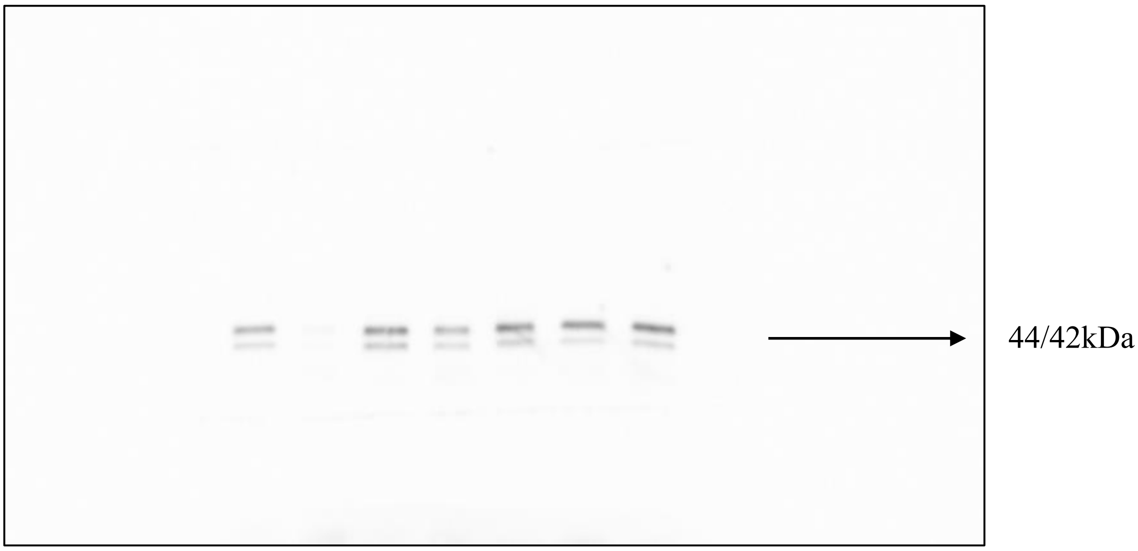

Figure 2: Full-length blots/gels of ERK ½

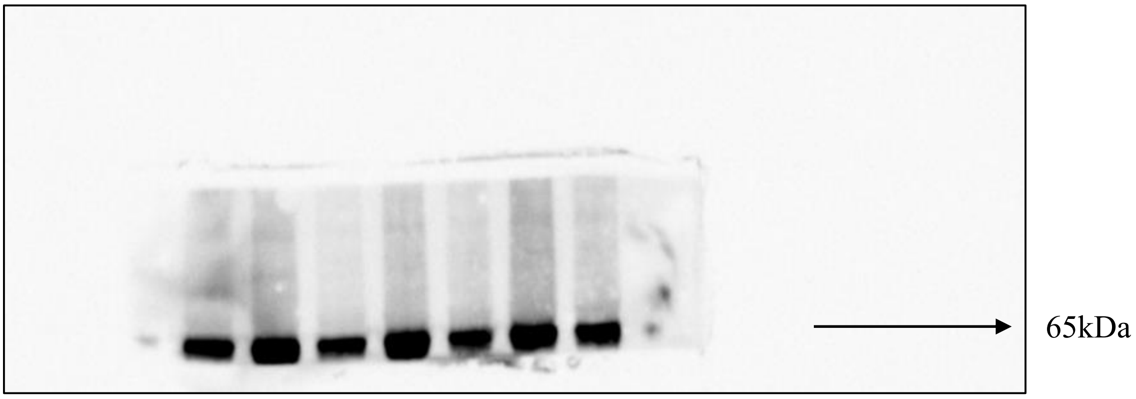

Figure 3: Full-length blots/gels of NF-κβ

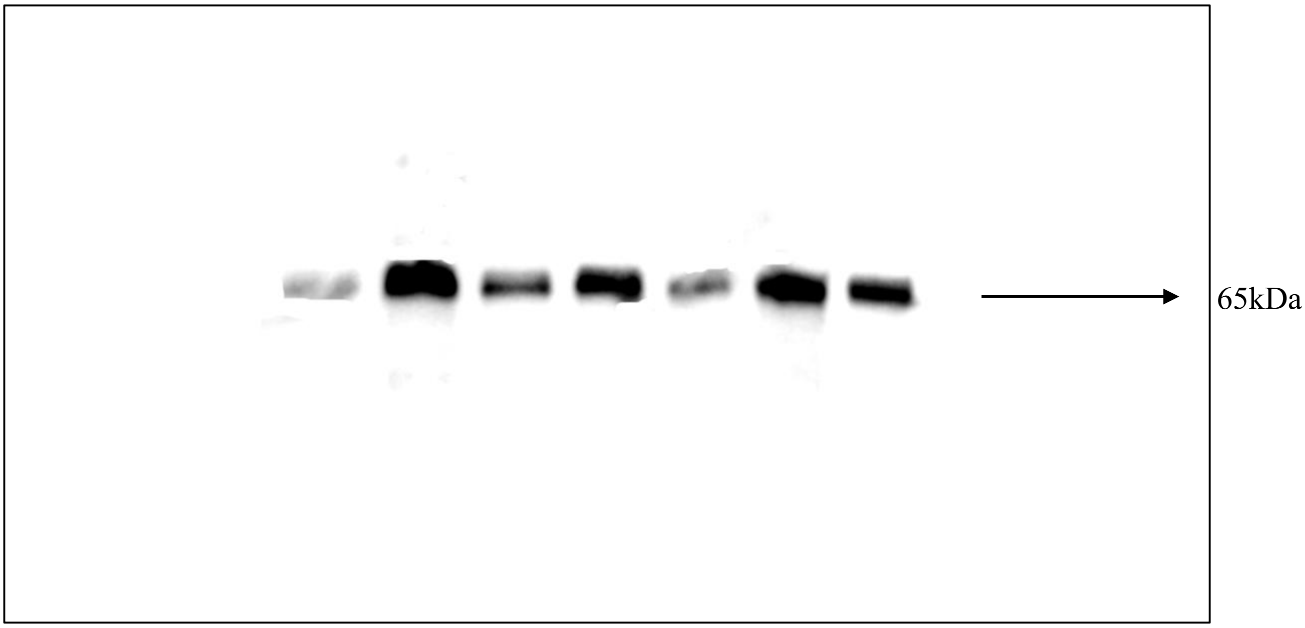

Figure 4: Full-length blots/gels of p-NF-κβ

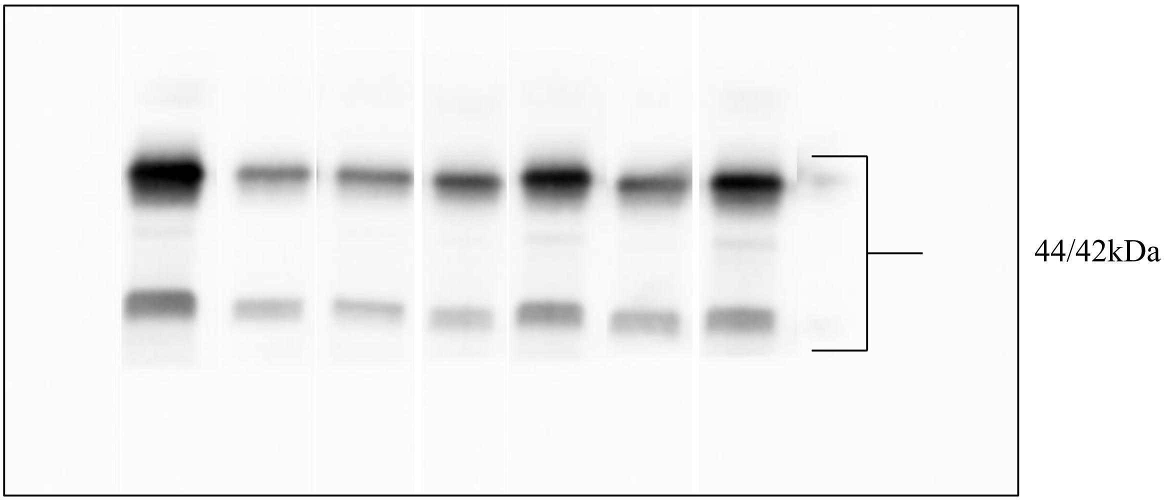

Figure 5: Full-length blots/gels of p-ERK ½ (Processed)

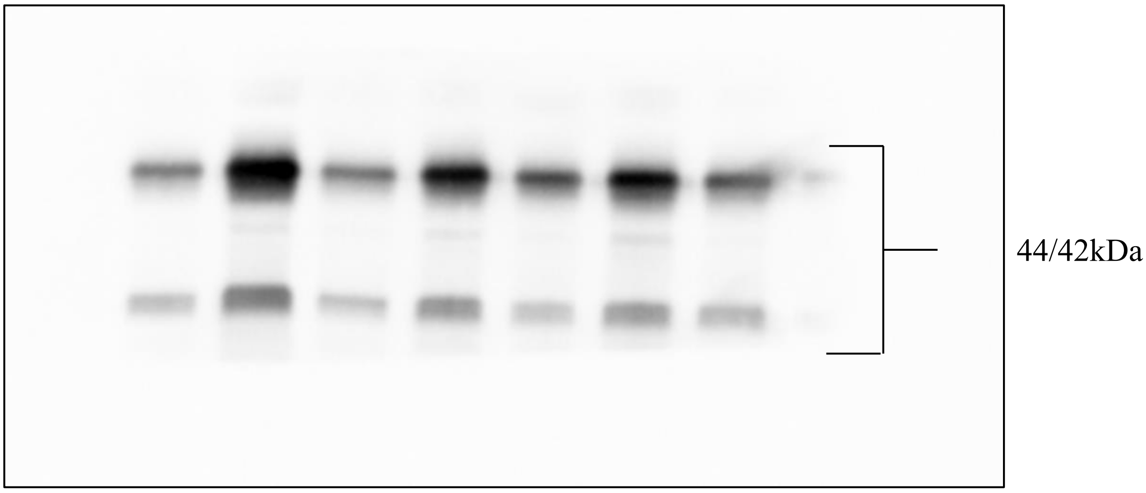

Figure 6: Full-length blots/gels of p-ERK ½ (Unprocessed)

Figure 6 is the unprocessed or original image of the p-ERK ½ however, in this image the sequences of test samples are not the same with other images. In **Figure 6** the sequences of test samples are as following:
C-4: Control 4th day, -ve C: negative control, C-16: Control 16th day, S-16: Standard 16th day, S-4: Standard 4th day, F-16: Formulation 16th day, F-4: Formulation 4th day

Therefore to maintain the similar sequence like other images, (such as -ve C: negative control, C-4: Control 4th day, C-16: Control 16th day, S-4: Standard 4th day, S-16: Standard 16th day, F-4: Formulation 4th day, F-16: Formulation 16th day) the processed image (**Figure 5**) has been provided for better understanding.

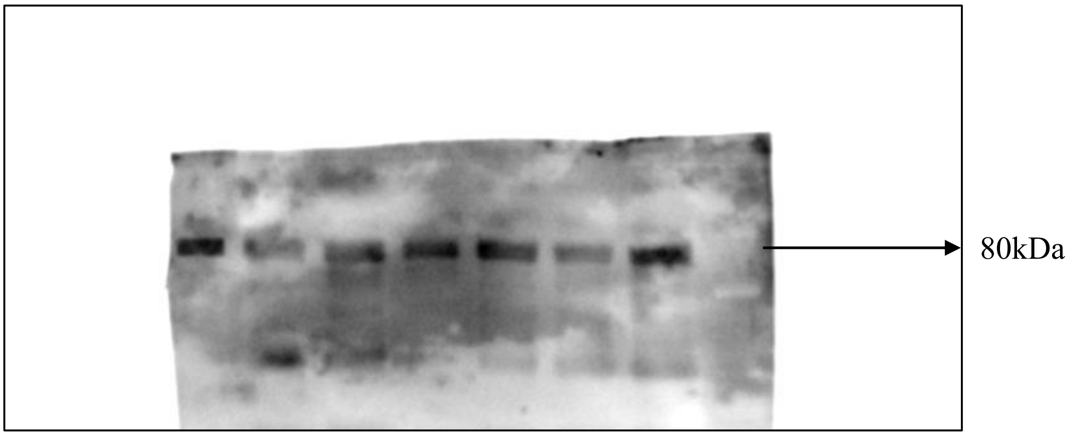

Figure 7: Full-length blots/gels of Trk-A

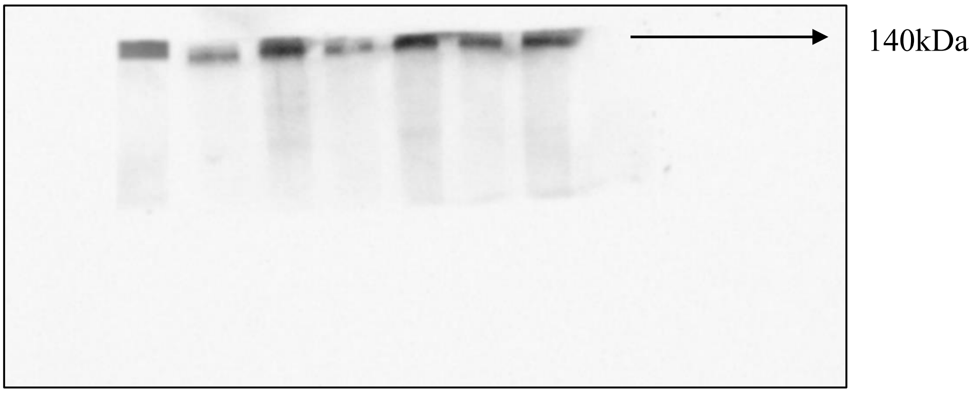

Figure 8: Full-length blots/gels of p-Trk-A

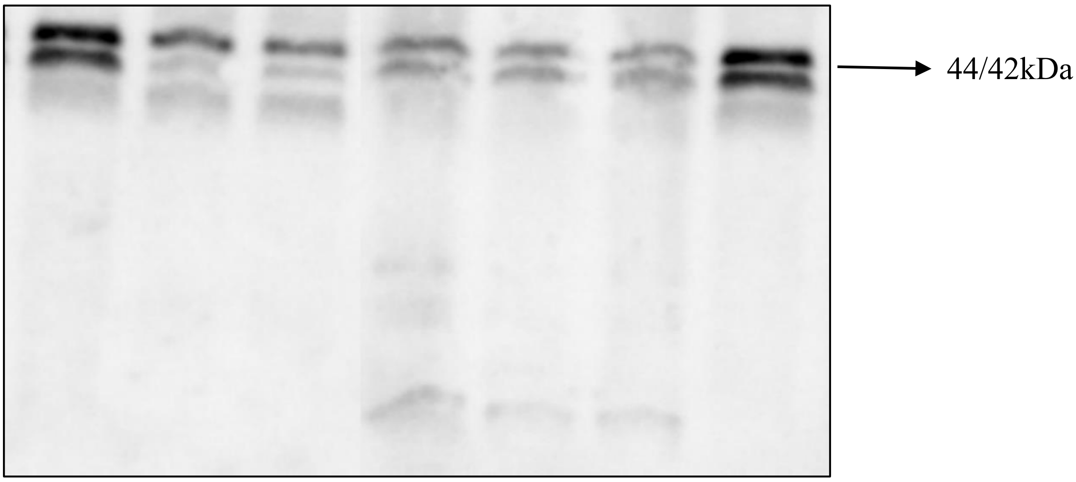

Figure: Replicate blot of ERK1/2

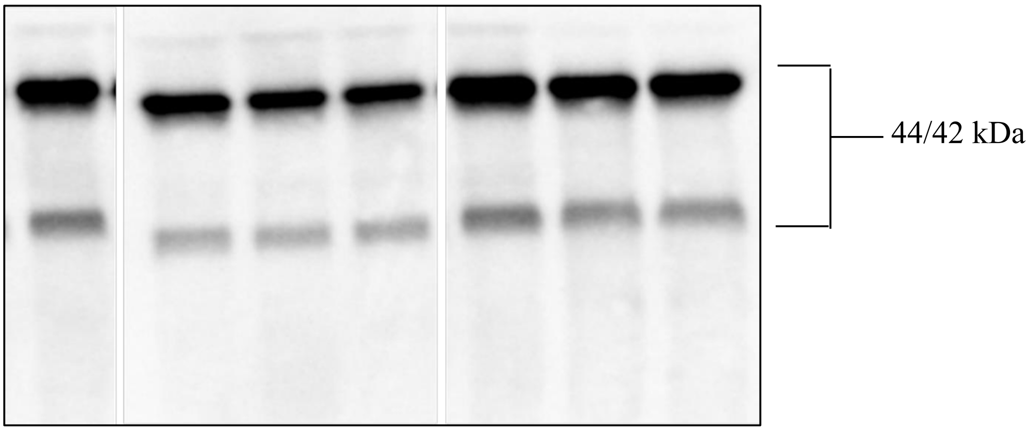

Figure: Replicate blot of p-ERK1/2

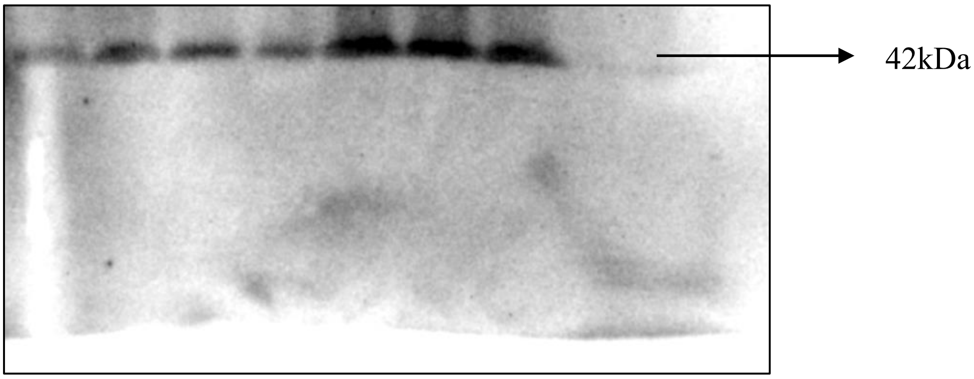

Figure: Replicate blot of β actin

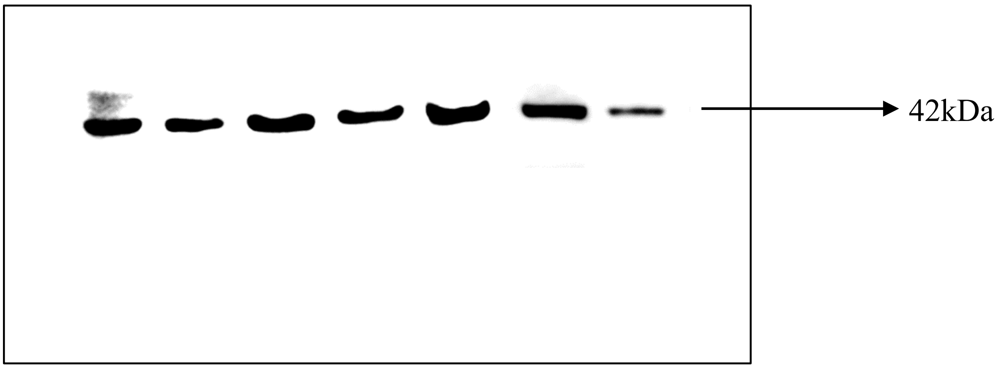

Figure: Replicate blot of β actin
